# Packed Red Blood Cell Transfusion as a Potential Treatment Option in COVID-19 Patients With Hypoxemic Respiratory Failure: A Case Report

**DOI:** 10.7759/cureus.8398

**Published:** 2020-06-01

**Authors:** Teshager Ejigu, Nikunjkumar Patel, Anuradha Sharma, Jagan Mohan Rao Vanjarapu, Vinod Nookala

**Affiliations:** 1 Internal Medicine, Community Medical Center, Toms River, USA; 2 Internal Medicine, Hackensack Meridian Health - Ocean Medical Center, Brick, USA

**Keywords:** covid-19, novel coronavirus, packed red blood cell transfusion, hemoglobin, red blood cells, hypoxemia, rbc, sars-cov-2

## Abstract

Severe acute respiratory syndrome coronavirus 2 (SARS-CoV-2) is the cause of coronavirus disease 2019 (COVID-19), an ongoing pandemic that started as an outbreak in China. The clinical picture varies from asymptomatic or mild cases to critically ill patients. Most of the critically ill patients present with hypoxia due to acute respiratory distress syndrome. These patients have a poor prognosis, especially in people with underlying co-morbidities. We present a case report of a 78-year-old male with multiple co-morbidities initially presenting with cardiac arrest and COVID-19 who showed drastic clinical improvement after he was administered a packed red blood cell transfusion. The patient was initially intubated for acute respiratory failure but was extubated on the second day as the patient’s respiratory status improved. Although this patient had multiple comorbidities, he did extremely well after he received a packed red blood cell transfusion. Recently, there is some evidence showing the effect of the novel coronavirus on hemoglobin levels. Poor clinical outcomes of critically ill patients are most likely due to the impaired gaseous exchange in the lungs in addition to the decreased oxygen-carrying capacity caused by the destruction of red blood cells. Currently, there is limited evidence available in this area and further research may help in developing effective treatment strategies.

## Introduction

A novel coronavirus was identified in December 2019 as the cause of an outbreak of acute respiratory illness, now known as coronavirus disease 2019 (COVID-19) in Wuhan, China. The virus that causes COVID-19 was named severe acute respiratory syndrome coronavirus 2 (SARS-CoV-2) [1]. This outbreak grew into an ongoing pandemic. The clinical picture of reported cases ranged from mild or asymptomatic cases to severe illnesses which cause significant mortality and morbidity [2-3]. While patients presenting with mild symptoms are reported to recover quickly, patients with severe symptoms, who generally present with severe respiratory symptoms, have far worse outcomes. Most of the patients with poor outcomes are older or have underlying comorbidities such as diabetes, hypertension, coronary heart disease, chronic lung conditions, cancers, or recent surgery, among others [4-7]. The management of COVID-19 is not yet clearly established and an increasing number of infections requiring ventilatory support is straining healthcare systems. According to data released by the Centers for Disease Control and Prevention from a group of approximately 2400 patients in the United States, hospitalization rates were 20.7-31.4%, ICU admission rates were 4.9-11.5%, and case fatality rates were 1.8-3.4% [8]. The severity of the disease and mortality increased with age [9]. Among critically ill patients, hypoxemic respiratory failure due to acute respiratory distress syndrome (ARDS) is the predominant finding. Most of the patients with severe features need either oxygen therapy or ventilation. To date, there is no standard treatment for COVID-19 that has been established, but medications such as hydroxychloroquine, azithromycin, antivirals, thiamine, vitamin-C, zinc, antivirals, and in some critically ill patients, convalescent plasma therapy have shown some beneficial effects [10]. We propose a newer treatment modality, packed red cell transfusion, that brings about drastic clinical improvement in a critically ill patient. Packed red blood cell transfusion, as in this patient's case, could become an effective adjuvant, especially in patients with hypoxic respiratory failure. This will, in turn, improve overall morbidity and mortality, and also decrease the burden on healthcare systems.

## Case presentation

A 78-year-old male with a past medical history of hypertension, diabetes mellitus, congestive heart failure with an ejection fraction of 20-25%, severe aortic stenosis, diverticulosis, chronic obstructive pulmonary disease (COPD), and anemia due to underlying gastrointestinal bleeding presented to the emergency department with shortness of breath and went into cardiac arrest. The patient attained return of spontaneous circulation following cardiopulmonary resuscitation in the emergency department. 

**Figure 1:**
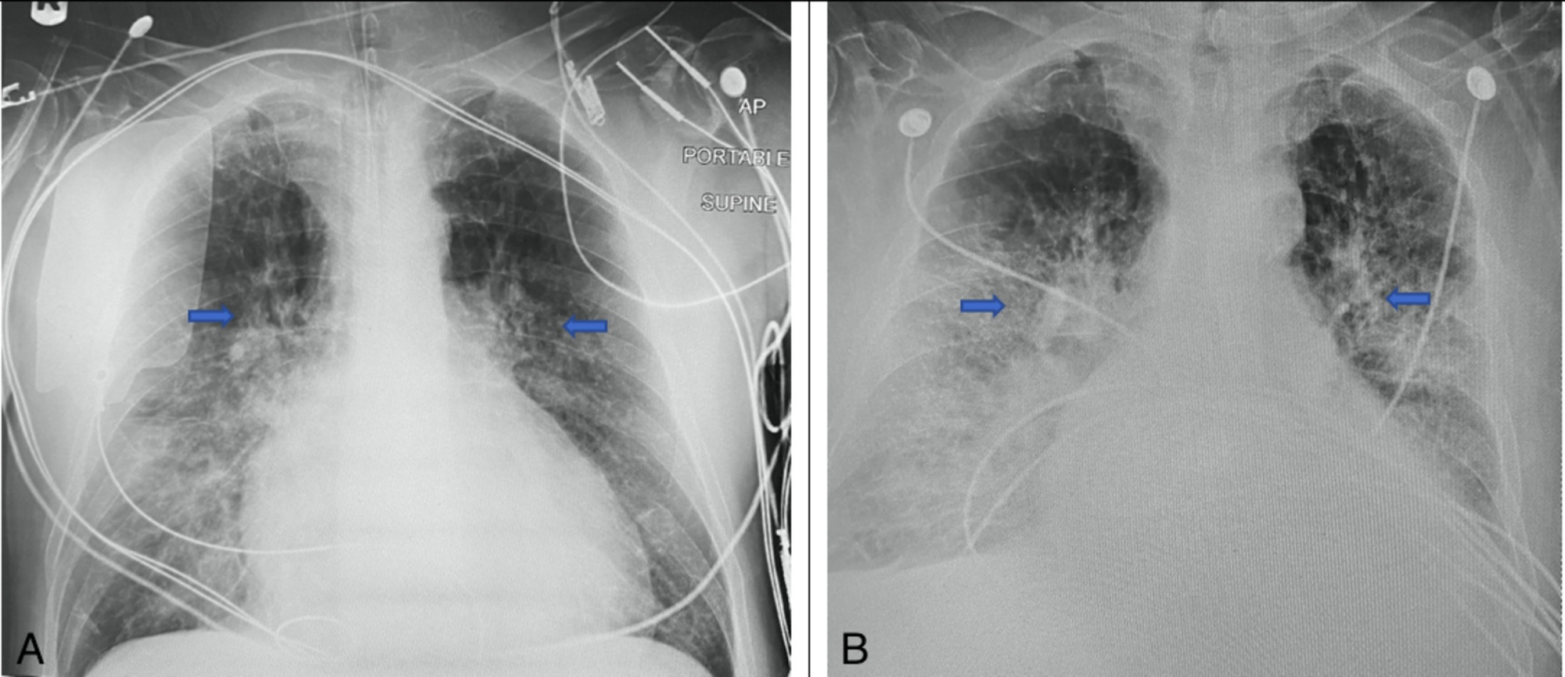
X-rays of chest from anteroposterior view from day 1 (A) and day 2 (B) respectively. The arrows in the above image indicate bilateral perihilar and infrahilar opacities with predominant involvement on the right side.

After he was stabilized, his vitals showed a temperature of 96.4° F, a pulse of 86 beats/minute, respiratory rate of 22 breaths/minute, blood pressure of 87/39 mm Hg, and oxygen saturation of 100%. On examination, he had bilateral 1+ pitting edema. His labs on the day of admission revealed a white blood cell count of 23.9 K/µL, a hemoglobin level of 4.5 mg/dL, hematocrit of 15.7 %, and platelet count of 458 K/µL. His blood urea nitrogen (BUN) test was 50 mg/dL with an elevated serum creatinine level of 2.16 mg/dL. The coagulation profile revealed prothrombin time of 17.5 and internalized normalized ratio (INR) of 1.46. His other laboratory investigations revealed lactic acid 3.6 mg/dL, troponin 19.6 ng/mL, AST 80 U/L, ALT 45 U/L, and alkaline phosphatase 74 U/L. His chest X-rays revealed bilateral perihilar, infrahilar, interstitial, and alveolar infiltrates on day 1 (Figure 1A) and day 6 (Figure 1B). The patient was intubated and mechanically ventilated for acute respiratory failure, was administered IV epinephrine for cardiogenic shock. Five units of packed red blood cells were transfused for severe anemia due to gastrointestinal blood loss. Later on the day of the presentation, he tested positive for SARS-CoV-2 by reverse transcriptase-polymerase chain reaction from his nasopharyngeal swab. Other tests for respiratory pathogens were negative. He was started on hydroxychloroquine, azithromycin, Vitamin C & zinc, thiamine, and melatonin. On day 2, the patient's respiratory status improved drastically and he was extubated and placed on a bilevel positive airway pressure (BiPAP) machine. On day 10, the patient was feeling significantly better and he was placed on a nasal cannula, with marked improvement in his clinical condition. He was clinically stable and discharged home on day 12.

## Discussion

Coronavirus disease 2019 (COVID-19) is an acute respiratory illness caused by SARS-CoV-2, a beta coronavirus that has caused an ongoing pandemic. COVID-19 has a wide clinical spectrum ranging from asymptomatic or mild cases to critically ill patients. Among them, critically ill patients were observed to have high rates of ARDS and a high risk of death [11]. In most hospitals, critically ill COVID-19 patients with ARDS are managed with mechanical ventilation. This is believed to recruit more alveoli, improve oxygenation, and improve transpulmonary pressures, and thus, a better chance to heal [12]. Mechanical ventilation also puts the patient at risk for various procedure-related hazards and increases the risk of contracting various infections for healthcare workers [12]. Most of the clinical manifestations of COVID-19 are thought to be caused by cytokine storm [13], leading to an exaggerated inflammatory response in the body. The virus is also known to attack red blood cells, thus decreasing the oxygen-carrying capacity. Understanding its effect on red blood cells may help in developing new therapeutic options for COVID-19 and decrease the number of intubations, as an increase in oxygen-carrying capacity will prevent the patient from going into a hypoxic state.

One published study cited the effect of the novel coronavirus on hemoglobin levels in COVID-19 patients. The hemoglobin levels of COVID-19 patients in this study group were significantly lower, especially in severely ill patients [14]. The decrease in hemoglobin levels in a critically ill patient with ARDS further worsens tissue oxygenation. A clear understanding of the virus’s effect on red blood cells could help in the development of better management strategies as maintaining the normal oxygen-carrying capacity is very important in patients with impaired pulmonary gas exchange due to ARDS.

The SARS-CoV-2 virus has an envelope, surface glycoproteins, and a nucleocapsid. Surface glycoproteins and viral open reading frames (ORF), namely, ORF8 with other proteins such as ORF10, ORF1ab, and ORF3a proteins are believed to attack the heme portion on the 1-beta chain of globin, and ORF8 protein binds to the porphyrin part of the heme [15]. This results in the destruction of heme, leading to less and less hemoglobin, thus affecting the oxygen-carrying capacity. This will cause decreased oxygen levels, compromising tissue oxygenation in the body.

Our patient presented with cardiac arrest and multiple comorbidities including COPD, congestive heart failure, and anemia due to ongoing gastrointestinal bleeding. He tested positive for SARS- CoV-2, with bilateral interstitial infiltrates on his chest X-ray. Since the patient presented with severe anemia, he was given packed red blood cells for anemia. The patient was intubated and mechanically ventilated for acute respiratory failure. On day 2, the patient's oxygen status improved and he was extubated. According to a study performed in a group of 27 patients in Seattle, the median duration of mechanical ventilation is around 10 days [16]. Since this patient presented with multiple comorbidities and a cardiac arrest, we initially had expected the patient to need ventilatory support for a longer duration. However, he improved drastically after he was given five units of packed red blood cells. With this in mind, we may need to consider increasing the threshold level of hemoglobin at which the patient needs packed red blood cells, especially in COVID-19 patients with hypoxic features.

## Conclusions

COVID-19 has a wide spectrum of presentations ranging from asymptomatic or mild symptoms to critically ill patients. Critically ill COVID-19 patients with ARDS have a poor prognosis, especially in the older age group. Poor clinical outcomes are probably caused by the impaired gaseous exchange in the lungs and decreased oxygen-carrying capacity due to the destruction of red blood cells. Transfusion of packed red blood cells may be a good therapeutic adjuvant in critically ill COVID-19 patients. The SARS-CoV-2 virus is thought to cause destruction of heme leading to a decrease in oxygen-carrying capacity, and clinicians should consider transfusion of packed red blood cells in critically ill patients with borderline low hemoglobin levels. More research in this area is needed to get a better understanding of the virus’s effects on red blood cells, and to develop effective treatment strategies.
